# Prediction model for gestational diabetes mellitus using the XG Boost machine learning algorithm

**DOI:** 10.3389/fendo.2023.1105062

**Published:** 2023-03-09

**Authors:** Xiaoqi Hu, Xiaolin Hu, Ya Yu, Jia Wang

**Affiliations:** ^1^ Department of Nursing, Yantian District People's Hospital, Shenzhen, Guangdong, China; ^2^ School of Basic Medical Sciences, Southern Medical University, Guangzhou, Guangdong, China; ^3^ Department of Nursing, Guangzhou First People's Hospital, Guangzhou, Guangdong, China; ^4^ Department of Nursing, Shenzhen Hospital of Southern Medical University, Shenzhen, Guangdong, China

**Keywords:** gestational diabetes mellitus, machine learning, prediction model, extreme gradient boosting, logistic regression

## Abstract

**Objective:**

To develop the extreme gradient boosting (XG Boost) machine learning (ML) model for predicting gestational diabetes mellitus (GDM) compared with a model using the traditional logistic regression (LR) method.

**Methods:**

A case–control study was carried out among pregnant women, who were assigned to either the training set (these women were recruited from August 2019 to November 2019) or the testing set (these women were recruited in August 2020). We applied the XG Boost ML model approach to identify the best set of predictors out of a set of 33 variables. The performance of the prediction model was determined by using the area under the receiver operating characteristic (ROC) curve (AUC) to assess discrimination, and the Hosmer–Lemeshow (HL) test and calibration plots to assess calibration. Decision curve analysis (DCA) was introduced to evaluate the clinical use of each of the models.

**Results:**

A total of 735 and 190 pregnant women were included in the training and testing sets, respectively. The XG Boost ML model, which included 20 predictors, resulted in an AUC of 0.946 and yielded a predictive accuracy of 0.875, whereas the model using a traditional LR included four predictors and presented an AUC of 0.752 and yielded a predictive accuracy of 0.786. The HL test and calibration plots show that the two models have good calibration. DCA indicated that treating only those women whom the XG Boost ML model predicts are at risk of GDM confers a net benefit compared with treating all women or treating none.

**Conclusions:**

The established model using XG Boost ML showed better predictive ability than the traditional LR model in terms of discrimination. The calibration performance of both models was good.

## Introduction

Gestational diabetes mellitus (GDM) is the most common metabolic complication to occur during pregnancy and is classed as a mild form of diabetes. It is normally diagnosed at 24–28 weeks’ gestation, and is characterized by hyperglycemia (1). The global prevalence of hyperglycemia during pregnancy is approximately 15.8%, and over 80% of cases are due to GDM (2). With the growth of the economy and the transition to a more sedentary lifestyle, the prevalence of GDM in Chinese women continues to increase, and ranges from 14.8% to 24.24% (3–5). Over time, China has loosened its fertility restrictions, most recently with the replacement of the two-child policy with the three-child policy. Thus, this increase in GDM prevalence can be attributed mainly to the rising rates of pregnant women who are of advanced maternal age.

Hyperglycemia brings about both short- and long-term outcomes, resulting in a significant impact on the health of both pregnant women and their offspring. Several studies in mothers have reported that GDM is associated with adverse pregnancy complications, including pre-eclampsia, the need for delivery by cesarean section, as well as type 2 diabetes and cardiovascular disease after delivery (6). GDM can also affect their offspring, being associated with a higher prevalence of macrosomia, shoulder dystocia, birth trauma, stillbirth, and, in later life, obesity and metabolic syndrome (7). According to the Developmental Origins of Health and Disease framework for GDM, exposure to intrauterine hyperglycemia before GDM screening at 24–28 weeks’ gestation is associated with the abnormal growth and development of the fetus (8). which includes smaller fetuses at 24 weeks’ gestation increased abdominal circumference growth rates (9), and hyperinsulinemia (6). Lifestyle interventions during early pregnancy can reduce the risk of GDM by 18%–62% (10, 11), but are not effective if initiated at a later stage (12). Thus, we concluded that a hysteretic diagnosis of GDM in the second or third trimester of pregnancy might lead to a narrow time frame for sufficient intervention. Therefore, it is imperative to establish a prediction model for women at risk of GDM to provide early intervention prior to the diagnosis of the condition at 24–28 weeks’ gestation.

There is accumulating evidence indicating that models based on multiple risk factors can improve predictive abilities (9). Machine learning (ML) algorithms, as an artificial intelligence technology, have the advantage of presenting high-dimensional predictors constructed to model relatively small datasets with reduced overfit, and demonstrate a powerful selflearning ability to find complex relationships between predictors (9, 13). As major predictors of GDM, demographic characteristics and clinical features contribute to improving the predictive ability of models combined with biomarkers (14, 15). Consequently, we aim to present the results of prediction models for GDM based on demographic characteristics, clinical features, and laboratory parameters to make full use of the available variables. In addition, we compare and evaluate the performance of ML and logistic regression (LR) models to show the advantages of each.

## Materials and methods

### Participants

This case–control study of pregnant women was conducted at the Shenzhen Hospital of the Southern Medical University, Shenzhen, China. Pregnant women were eligible to participate in the study if they met all of the following inclusion criteria: (1) they were aged ≥ 18 years; (2) they had undergone all routine antenatal assessments; (3) they had taken a 75-g oral glucose tolerance test (OGTT) at 24–28 weeks’ gestation; and (4) they were willing to participate in this study and to sign the informed consent form. The exclusion criteria were as follows: (1) pre-existing type 1 or type 2 diabetes; (2) a history of severe diseases, such as hypertension or heart disease; and (3) taking medications affecting insulin and blood glucose levels.

### Data collection

Information on participants’ demographic characteristics was collected by using a structured questionnaire. Clinical features and laboratory parameters in the first trimester were collected from the hospital’s electronic medical record system (EMRS).

### Diagnosis of GDM

GDM was diagnosed at 24–28 weeks’ gestation when any one of the 75-g OGTT values met or exceeded 5.1 mmol/L at 0 h, 10.0 mmol/L at 1 h, and 8.5 mmol/L at 2 h, in accordance with the recommendations set out at the International Association of Diabetes and Pregnancy Study Groups Consensus Panel 2010 (IADPSG).

### Statistical analysis

All analyses were performed using IBM^®^ SPSS^®^ Statistics version 26.0 software (IBM Corporation, Armonk, NY, USA). Continuous variables of two groups were expressed as means and standard deviations, and analyzed by Student’s *t*-test for normally distributed variables. Categorical variables were described as frequencies (percentages), and evaluated by a chi-squared test. Test results with a *p*-value of less than 0.05 were considered statistically significant. Results from these tests, clinically relevant findings, and previous literature were used to preliminarily screen the set of variables for potentially meaningful predictors of GDM. Multiple imputations were used to deal with missing data, to avoid selection bias. The prediction model using LR was carried out in R (The R Foundation, Vienna, Austria) using the rms package, and XG Boost ML was carried out by R package (XG Boost, XG Boost Explainer, and MLR).

### Prediction models

In this study, we included variables with a *p*-value of < 0.05 in the univariate analysis, whereas variables indicated in previous literature and clinically meaningful variables were included in the LR analysis (stepwise). ML can present novel or complex combinations of multidomain variables, and also has features that weigh variable importance and reduce overfit (16). Therefore, we incorporated all variables of the univariate analysis into the model using XG Boost ML.

The model for GDM, trained on the training set, was validated in the testing set with the optimal hyperparameters using 10-fold cross-validation.

### Model evaluation

The discrimination of the models was assessed using the receiver operating characteristic (ROC) curves and the area under the ROC curve (AUC). The calibration plots and the Hosmer–Lemeshow (HL) test were used to evaluate the calibration of each model. Decision curve analysis (DCA) was introduced to evaluate the clinical use of the models.

## Results

### Participant characteristics

In total, 925 pregnant women were included in this study (735 in the training set; 190 in the testing set). The alternative 33 variables were collected for each pregnant woman. **Table 1** shows the univariate analysis of the demographic characteristics, clinical features, and laboratory parameters of participants with GDM (cases) and participants without GDM (controls) in the training set. Participants with GDM were significantly older and had higher pre-pregnancy body mass index (BMI) and mean arterial pressure (MAP) than participants without GDM. The average time since the last pregnancy was also longer in this group than in the control group. The percentage of women who had previously GDM and the number with a family history of diabetes mellitus were also significantly higher in the GDM group, but participants in this group were also markedly younger at menarche than those in the non-GDM group (all p-values were < 0.05). Laboratory parameters, including platelet count, white blood cell count, and the levels of glucose in urine, ketone in urine, alanine aminotransferase, thyroid hormone T_3_, fasting plasma glucose, and glycated hemoglobin (HbA_1c_), were also higher in women with GDM than in control participants. The demographic characteristics, clinical features, and laboratory parameters of participants in the training and testing sets are compared in **Table 2**. Good consistency in the data between the training data set and the testing data set is shown for the majority of the variables.

**Table 1:** Demographic characteristics, clinical features, and laboratory parameters of participants with GDM and non-GDM control participants in the training set.

Variable	GDM (*N *= 147)	Non-GDM (*N* = 588)	*p*-value
Demographic characteristics			
**Age (years)**	32.068 ± 4.208	30.005 ± 4.027	0.000^*^
**Occupation, *n* (%)**			0.254
None/homemaker	43 (29.25%)	145 (24.66%)	
Working	104 (70.75%)	443 (75.34%)	
**Time spent in education (years), *n* (%)**			0.705
< 12	18 (12.24%)	83 (14.12%)	
12–16	117 (79.59%)	466 (79.25%)	
> 16	12 (8.16%)	39 (6.63%)	
**Smoking, *n* (%)**	4 (2.72%)	11 (1.87%)	0.514
**Alcohol consumption, *n* (%)**	33 (22.45%)	179 (30.44%)	0.056
Clinical features, *n* (%)			
**Gravidity**			0.109
1	54 (36.73%)	259 (44.05%)	
≥ 2	93 (63.27%)	329 (55.95%)	
**Parity, *n* (%)**			0.193
0	76 (51.70%)	339 (57.65%)	
≥ 1	71 (48.30%)	249 (42.35%)	
Menarche age (years)	13.381 ± 1.411	13.536 ± 1.471	0.000^*^
Time since last pregnancy (years)	2.8027 ± 3.309	1.9354 ± 2.637	0.001^*^
Pre-pregnancy BMI (kg/m^2^)	21.681 ± 3.024	20.630 ± 2.582	0.000^*^
MAP (mmHg)	80.896 ± 8.822	78.641 ± 7.735	0.002^*^
Previous GDM, *n* (%)	36 (24.49%)	18 (3.06%)	0.000^*^
Previous macrosomia, *n* (%)	2 (1.36%)	8 (1.36%)	1.000
Polycystic ovary syndrome, *n* (%)	9 (6.12%)	21 (3.57%)	0.162
Family history of diabetes mellitus, *n* (%)	21 (14.29%)	51 (8.67%)	0.041^*^
Laboratory parameters			
Routine blood tests			
Hemoglobin (g/L)	123.232 ± 12.314	122.238 ± 11.076	0.086
Red blood cell count (× 10^12^/L)	4.147 ± 0.452	4.123 ± 0.452	0.061
Platelet count (× 10^9^/L)	244.612 ± 59.113	231.952 ± 54.730	0.014^*^
White blood cell count (×10^9^/L)	8.815 ± 2.240	8.408 ± 2.044	0.031^*^
Routine urine and renal function tests			
Urine specific gravity	1.019 ± 0.007	1.020 ± 0.008	0.075
Urine pH	6.643 ± 0.685	6.643 ± 0.690	0.417
Glucose in urine, *n* (%)	12(8.16%)	22(3.74%)	0.022^*^
Ketones in urine, *n* (%)	36(24.49%)	99(16.84%)	0.032^*^
Uric acid	64.295 ± 5.339	60.752 ± 2.518	0.086
Hepatic function tests			
Total bilirubin (μmol/L)	7.159 ± 3.255	7.072 ± 3.043	0.714
ALT (U/L)	14.256 ± 12.050	12.063 ± 7.540	0.008^*^
AST (U/L)	16.489 ± 7.023	15.721 ± 5.097	0.131
Total protein (g/L)	69.842 ± 5.304	69.445 ± 5.104	0.450
Thyroid function tests			
Thyroid-stimulating hormone (mIU/L)	1.598 ± 1.364	1.750 ± 1.415	0.677
Thyroid hormone T_3_ (nmol/L)	3.196 ± 1.784	3.010 ± 0.647	0.017^*^
Thyroid hormone T_4_ (nmol/L)	1.383 ± 0.586	1.396 ± 0.869	0.456
Glycemic test			
Fasting plasma glucose (mmol/L)	4.659 ± 0.426	4.562 ± 0.377	0.000^*^
HbA_1c_ (%)	5.225 ± 0.354	5.045 ± 0.315	0.004^*^

*p < 0.05.

ALT, alanine aminotransferase; AST, aspartate aminotransferase; BMI, body mass index; GDM, gestational diabetes mellitus; HbA_1c_, glycated hemoglobin; MAP, mean arterial pressure.

**Table 2:** Demographic characteristics, clinical features, and laboratory parameters of the training and testing sets.

Variables	Training set (*N* = 735)	Testing set (*N* = 190)	*p*-value
Demographic characteristics			
**Age (years)**	30.418 ± 4.144	29.474 ± 3.590	0.004^*^
**Occupation, *n* (%)**			0.255
None/homemaker	188 (25.578%)	41 (5.578%)	
Working	547 (74.422%)	149 (20.272%)	
**Time spent in education (years), *n* (%)**			0.125
< 12	101 (13.741%)	19 (2.585%)	
12–16	583 (79.320%)	151 (20.544%)	
> 16	51 (6.939%)	20 (2.721%)	
**Smoking), *n* (%)**	15 (2.041%)	4 (0.544%)	0.955
**Alcohol consumption**	212 (28.844%)	63 (8.571%)	0.246
Clinical features), *n* (%)			
**Gravidity**			0.117
1	313 (42.585%)	92 (12.517%)	
≥ 2	422 (57.415%)	96 (13.061%)	
**Parity**			0.032^*^
0	415 (56.463%)	123 (16.735%)	
≥ 1	320 (43.537%)	66 (8.980%)	
Menarche age (years)	13.505 ± 1.460	13.405 ± 1.724	0.421
Time since last pregnancy (years)	2.109 ± 2.803	1.739 ± 2.640	0.102
Pre-pregnancy BMI (kg/m^2^)	20.841 ± 2.707	20.966 ± 2.971	0.579
MAP (mmHg)	79.092 ± 8.009	81.422 ± 8.656	0.001^*^
Previous GDM, *n* (%)	54 (7.347%)	11 (1.497%)	0.454
Previous macrosomia, *n* (%)	10 (1.361%)	0 (0.000%)	0.106
Polycystic ovary syndrome, *n* (%)	30 (4.082%)	12 (1.633%)	0.187
Family history of diabetes mellitus, *n* (%)	72 (9.796%)	16 (2.177%)	0.565
Laboratory parameters			
Routine blood tests			
Hemoglobin (g/L)	122.437 ± 11.333	123.284 ± 10.072	0.348
Red blood cell count (× 10^12^/L)	4.128 ± 0.452	4.105 ± 0.444	0.528
Platelet count (× 10^9^/L)	234.489 ± 55.823	243.351 ± 54.367	0.050
White blood cell count (× 10^9^/L)	8.489 ± 2.090	8.795 ± 2.023	0.071
Routine urine and renal function tests			
Urine specific gravity	1.020 ± 0.008	1.017 ± 0.009	0.000^*^
Urine pH	6.643 ± 0.689	6.589 ± 0.679	0.333
Glucose in urine	1.112 ± 0.585	1.048 ± 0.317	0.155
Ketone in urine	1.457 ± 1.104	1.462 ± 1.106	0.954
Uric acid level	234.818 ± 61.470	215.842 ± 52.786	
Hepatic function tests			
Total bilirubin (μmol/L)	7.089 ± 61.470	7.309 ± 4.158	0.417
ALT (U/L)	12.502 ± 8.667	12.336 ± 6.579	0.806
AST (U/L)	15.874 ± 5.539	16.666 ± 3.611	0.062
Total protein (g/L)	69.524 ± 5.143	70.079 ± 5.133	0.185
Thyroid function tests			
Thyroid-stimulating hormone(mIU/L)	1.720 ± 1.405	1.847 ± 1.902	0.305
Thyroid hormone T_3_ (mmol/L)	3.047 ± 0.987	3.199 ± 0.898	0.054
Thyroid hormone T_4_ (mmol/L)	1.393 ± 0.820	1.354 ± 0.774	0.554
Glycemic test			
Fasting plasma glucose (mmol/L)	4.582 ± 0.389	4.369 ± 0.665	0.000^*^
HbA_1c_ (%)	5.081 ± 0.331	5.284 ± 0.318	0.000^*^

*p < 0.05.

ALT, alanine aminotransferase; AST, aspartate aminotransferase; GDM, gestational diabetes mellitus; HbA_1c_, glycated hemoglobin; MAP, mean arterial pressure.

### Predictors of models

**Figure 1:**
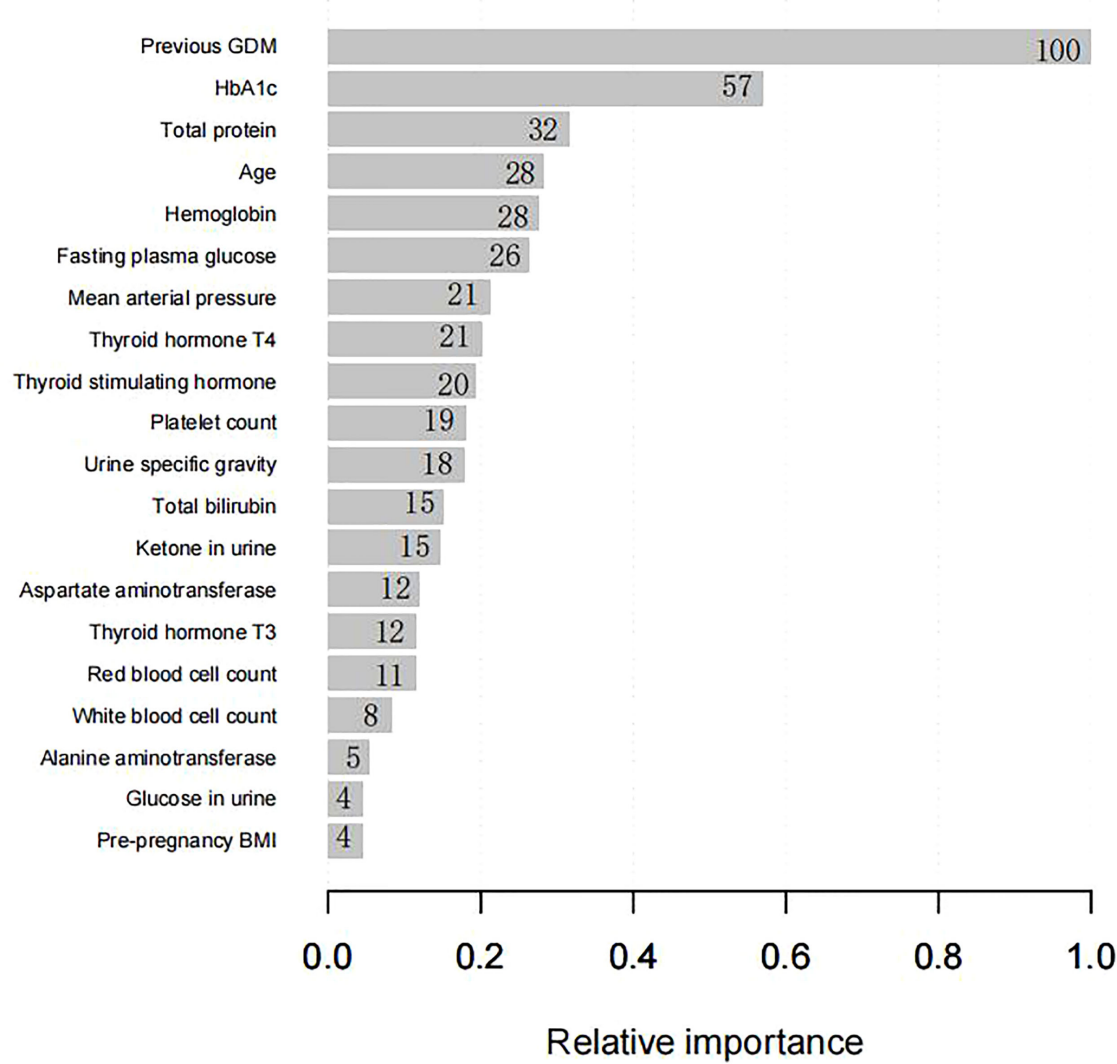
The relative importance of the 20 variables included in the XG Boost ML model for GDM in the training set. BMI, body mass index; GDM, gestational diabetes mellitus; HbA_1c_, glycated hemoglobin; XG Boost ML, extreme gradient boosting (XG) machine learning (ML).

Four predictors, previous GDM, age, HbA_1c_ level, and MAP, were used to construct the predictive model using LR (**Table 3**). Twenty predictors were finally included to build the model using XG Boost ML. **Figure 1** shows the relative importance of the 20 variables included in the predictive model for GDM using XG Boost ML.

**Table 3:** Four predictors included in the model using stepwise LR in the training set.

Variable	β	SE	*p*-value	OR	95% CI
Age (years)	0.096	0.024	0.000	1.101	1.052 to 1.154
Previous GDM	2.057	0.321	0.000	7.822	4.172 to 14.666
MAP (mmHg)	0.029	0.012	0.020	1.029	1.005 to 1.054
HbA_1c_ (%)	1.301	0.335	0.000	3.672	1.903 to 7.083
Constant	–13.542	2.045	0.000	0.000	0.000 to 0.000

LR, logistic regression; OR, odds ratio.

### Accuracy of prediction models

**Figure 2:**
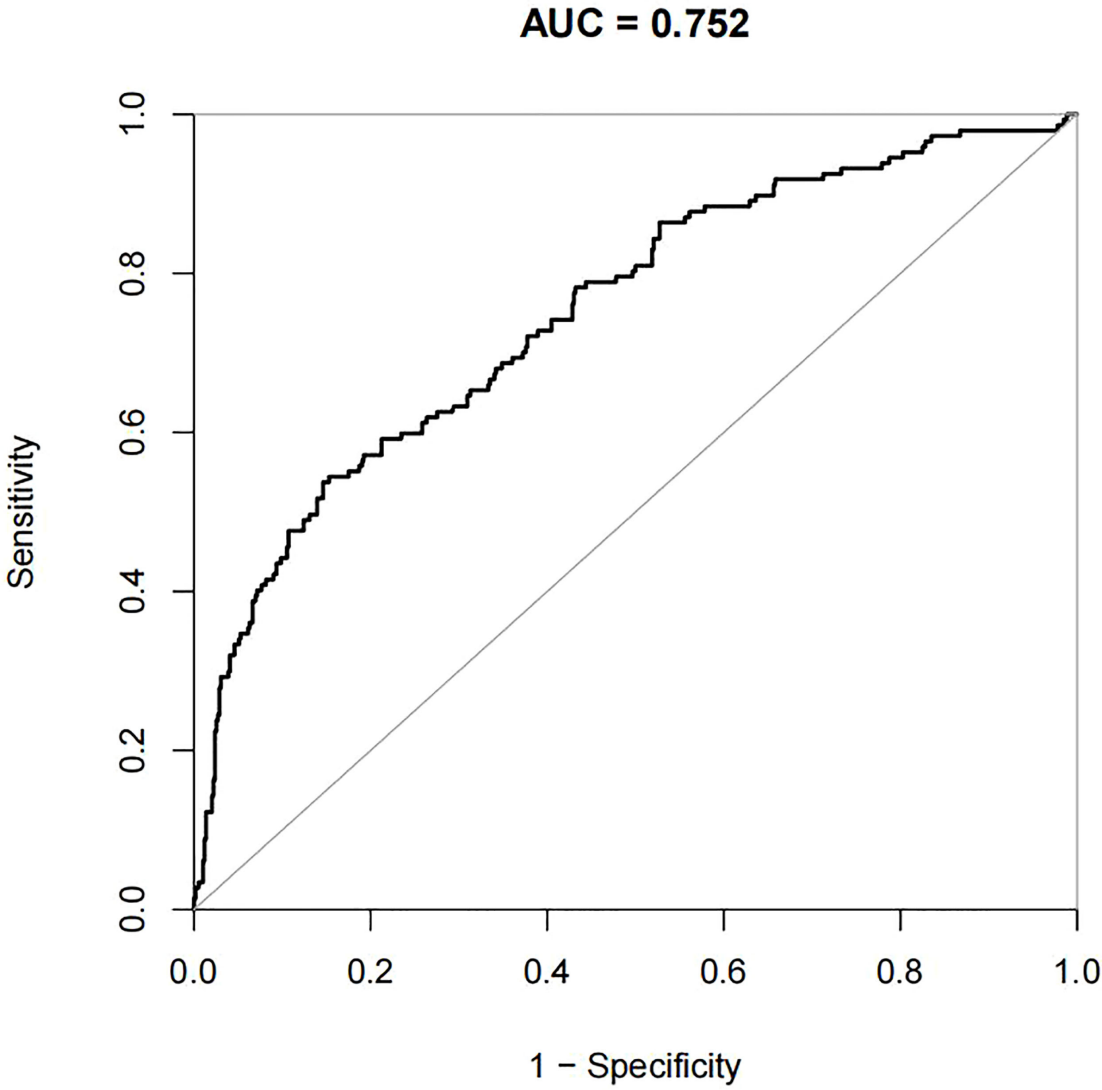
The AUC of the prediction model for GDM by stepwise LR. AUC, area under the receiver operating characteristic curve; GDM, gestational diabetes mellitus; LR, logistic regression.

**Figure 3:**
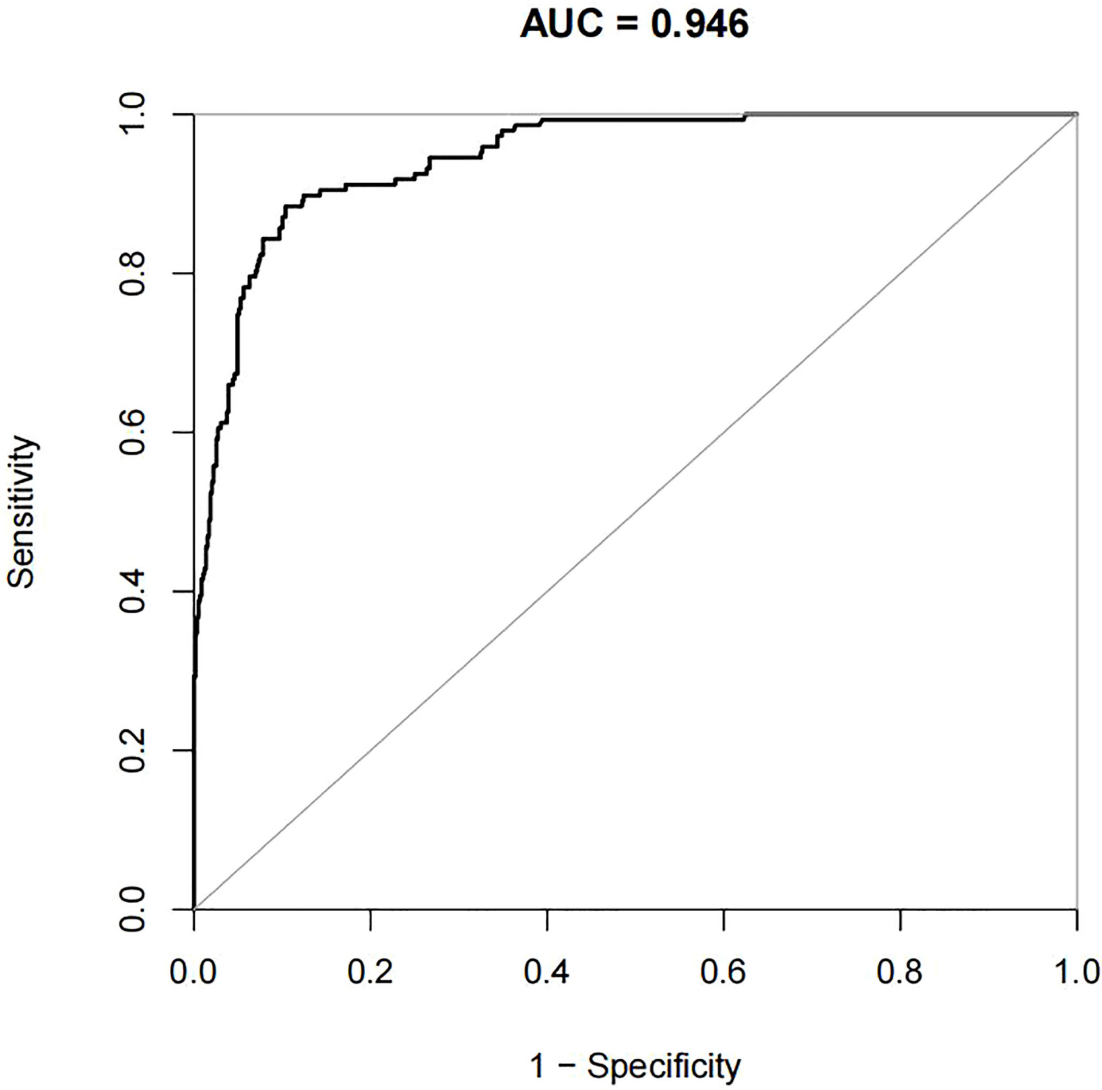
The AUC of the prediction model for GDM by XG Boost ML. AUC, area under the receiver operating characteristic curve; GDM, gestational diabetes mellitus; XG Boost ML, extreme gradient boosting (XG) machine learning (ML).

For the data from the training set, the AUC of the prediction model for GDM using stepwise LR is 0.752, whereas the AUC of the model using XG Boost ML is 0.946; these are shown in **Figures 2**, **3**, respectively. The accuracy of the two models for the data from the training set is 0.786 and 0.875, respectively. The specificity of the model using XG Boost ML was higher than that of the model using traditional LR for the data from both the training and testing sets. However, the sensitivity of the model using XG Boost ML was lower than that of the model using traditional LR, as shown clearly in **Table 4**.

**Table 4:** Accuracy of the four prediction models.

		Accuracy (95% Cl)	AUC (95% Cl)	Cut-off point	Youden’s index	Sensitivity	Specificity	Positive predictive value	Negative predictive value
Training set (*N* = 735)	Model using stepwise logistic regression	0.786	0.752 (0.706 to 0.797)	0.240	0.391	0.544	0.847	0.471	0.881
	Model using XG Boost ML	0.875 (0.849 to 0.898)	0.946	0.500	0.783	0.408	0.992	0.923	0.870
Testing set (*N* = 190)	Model using stepwise logistic regression	0.842	0.745 (0.648 to 0.842)	0.310	0.433	0.500	0.922	0.600	0.888
	Model using XG Boost ML	0.837 (0.777 to 0.886)	0.750	0.518	0.697	0.250	0.974	0.692	0.848

AUC, area under the receiver operating characteristic curve; XG Boost ML, extreme gradient boosting (XG) machine learning (ML).

### Calibration of different models

**Figure 4:**
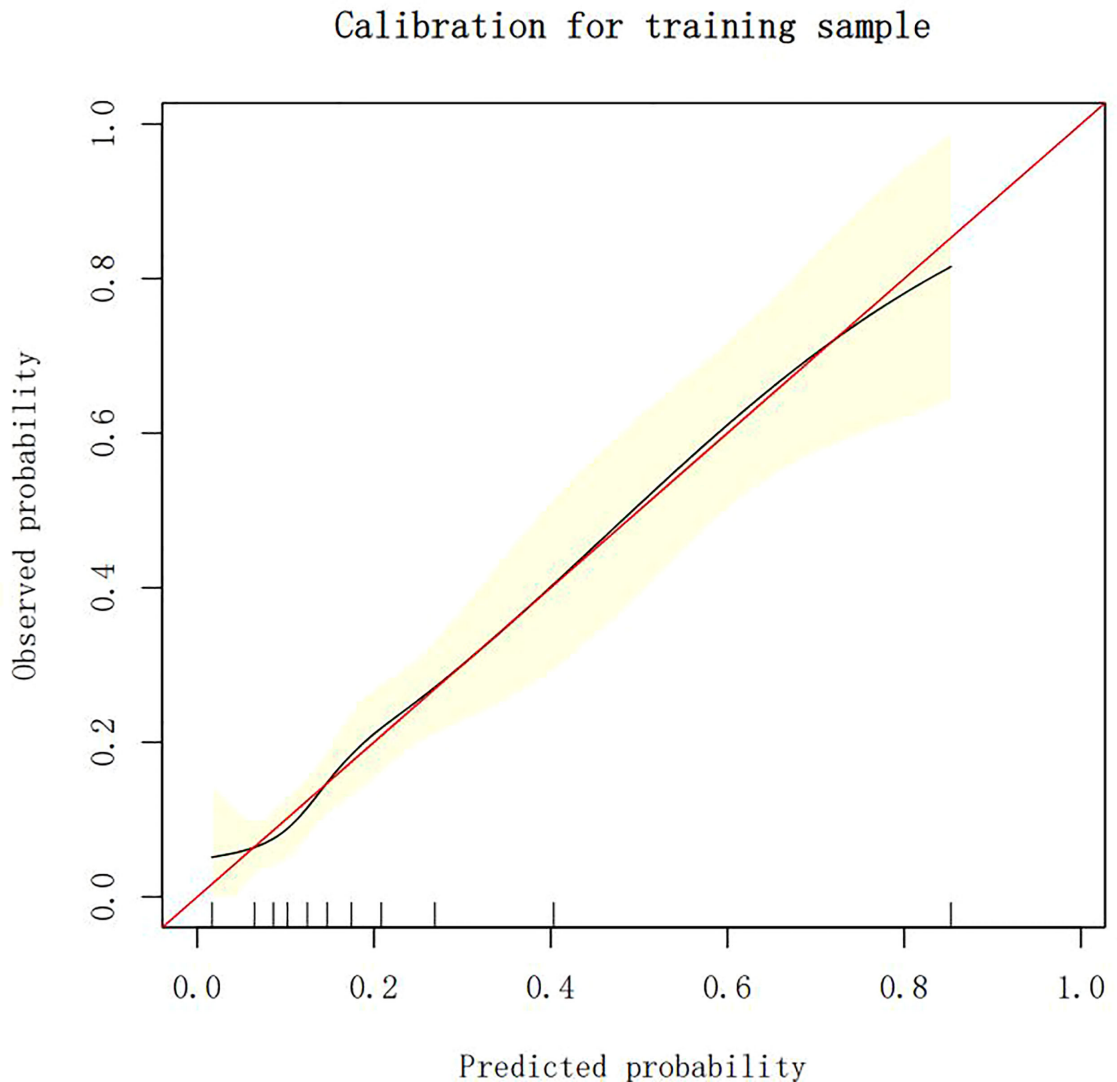
The calibration plots of the training set by LR. LR, logistic regression.

**Figure 5:**
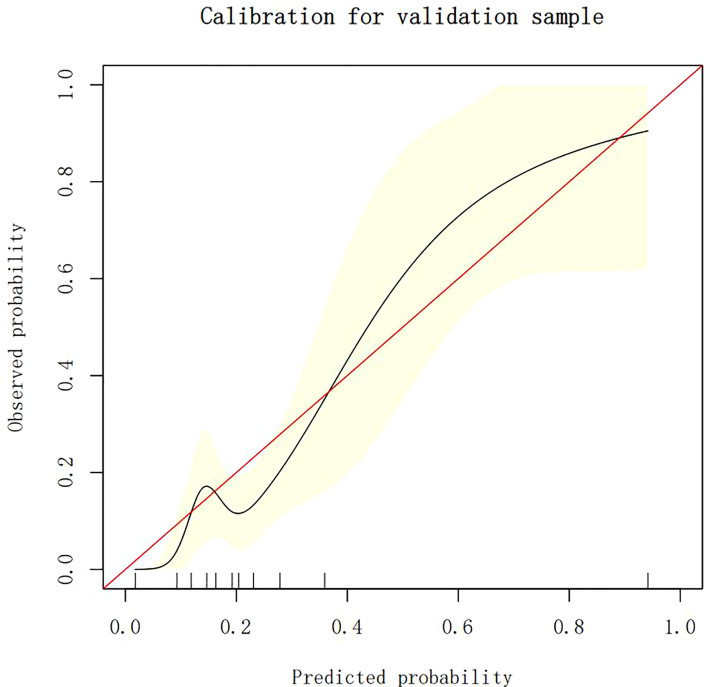
The calibration plots of the testing set by LR. LR, logistic regression.

**Figure 6:**
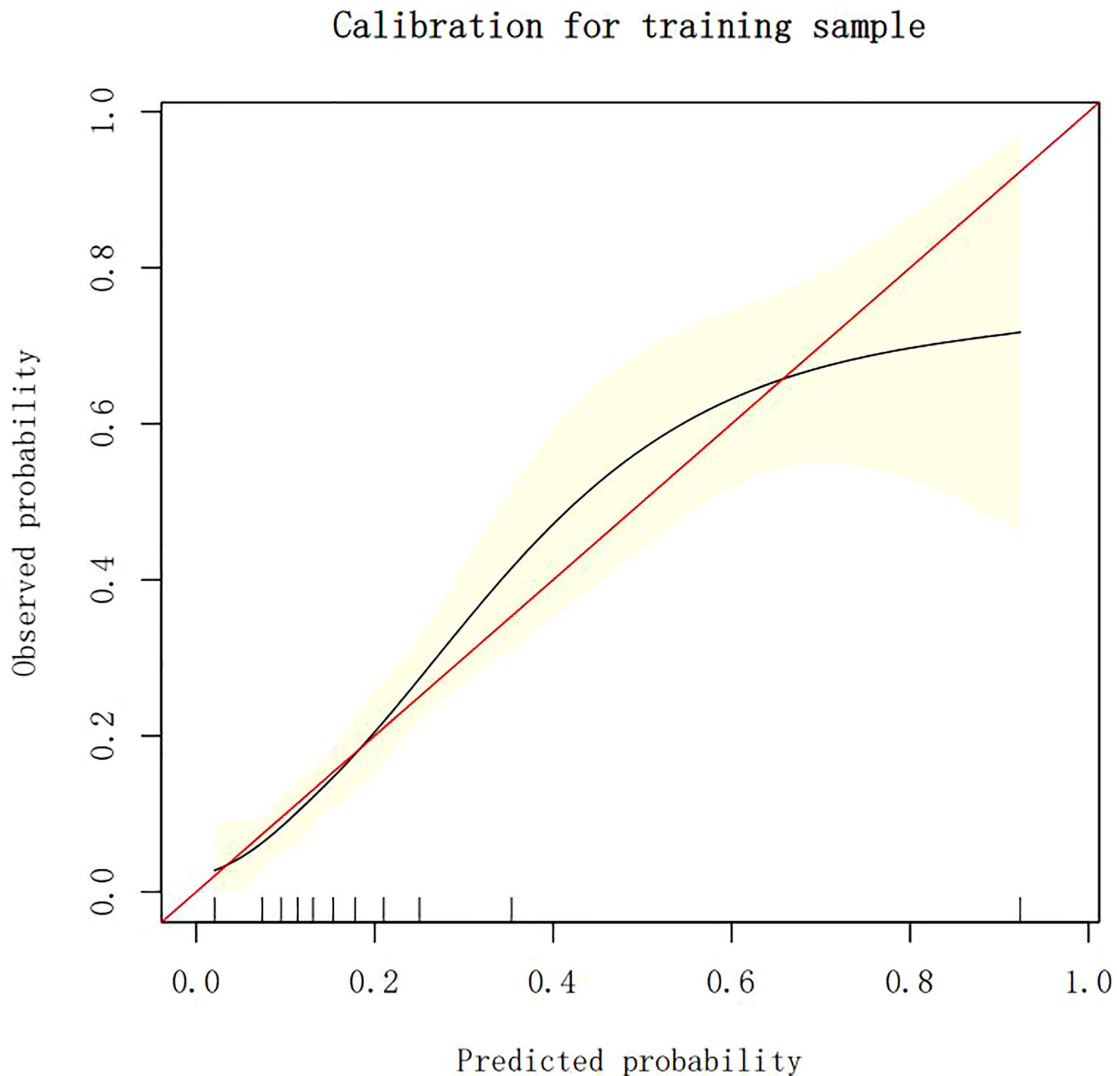
The calibration plots of the training set by XG Boost ML. XG Boost ML, extreme gradient boosting (XG) machine learning (ML).

**Figure 7:**
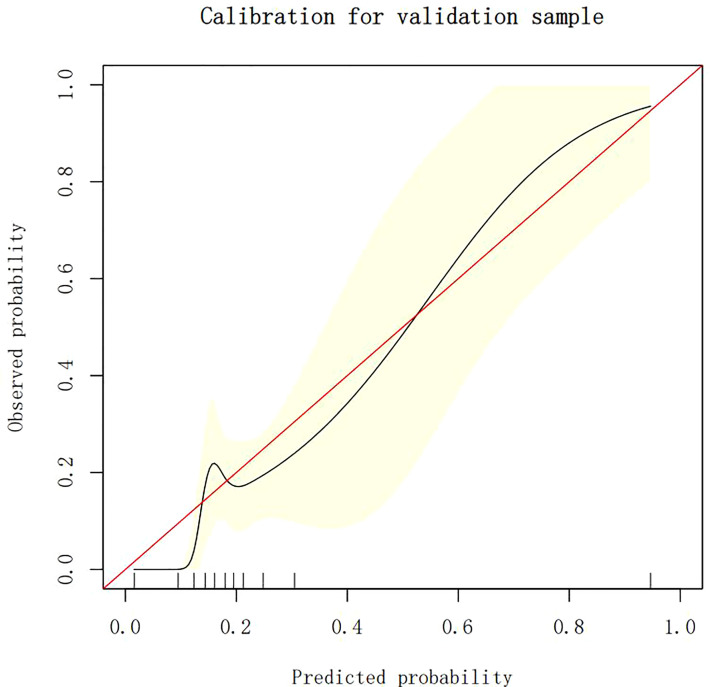
The calibration plots of the testing set by XG Boost ML. XG Boost ML, extreme gradient boosting (XG) machine learning (ML).

The calibration plots demonstrate the consistency between the predicted values and the real outcomes, which are shown in **Figures 4**–**7**. The Hosmer–Lemeshow (HL) test *p*-values were 0.288 and 0.402 for the training set and testing sets, respectively, in the model using LR, and 0.831 and 0.556 for the training set and testing sets, respectively, in the model using XG Boost ML.

### Clinical use

**Figure 8:**
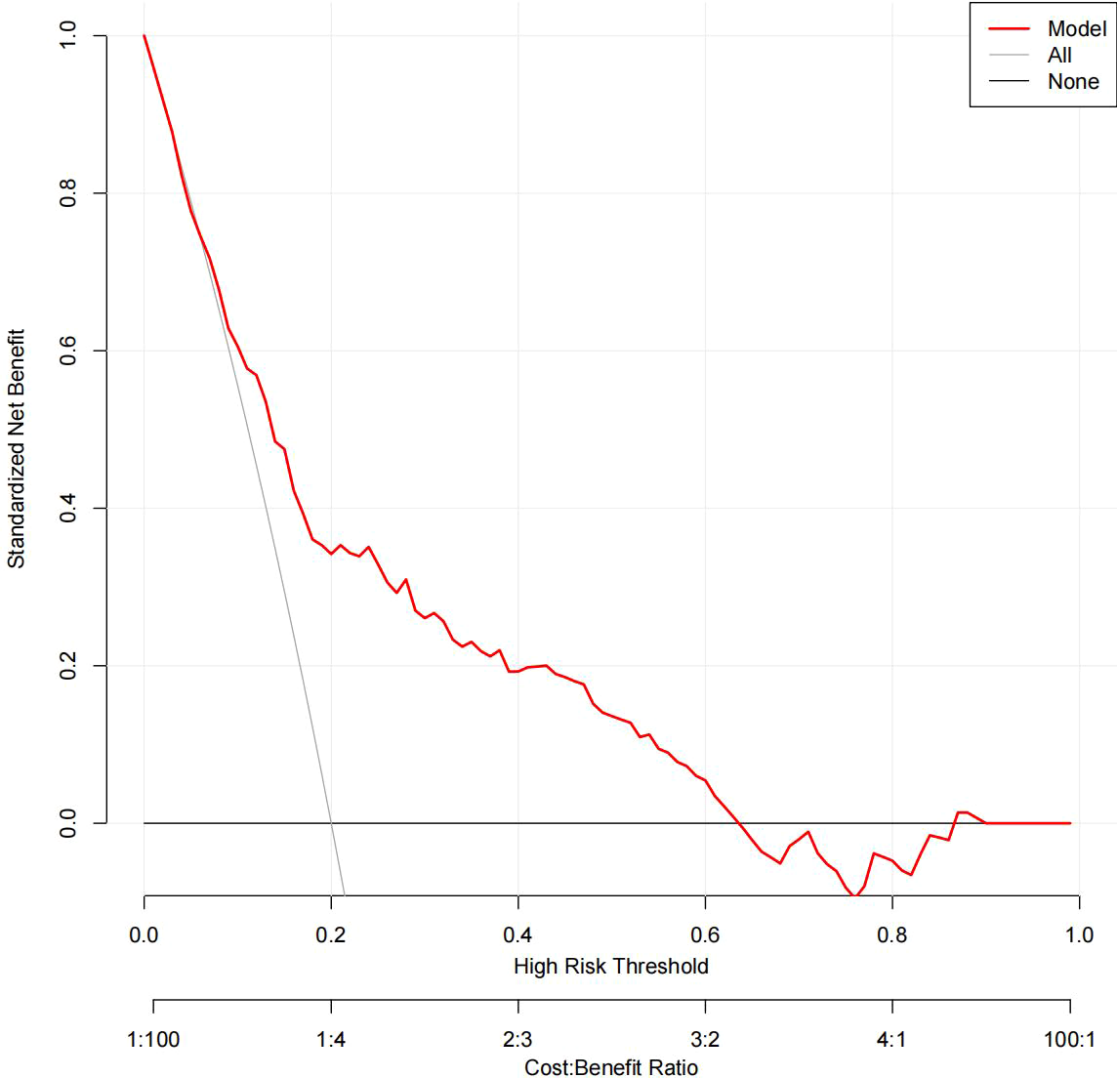
The DCA of the model using LR. DCA, decision curve analysis; LR, logistic regression.

**Figure 9:**
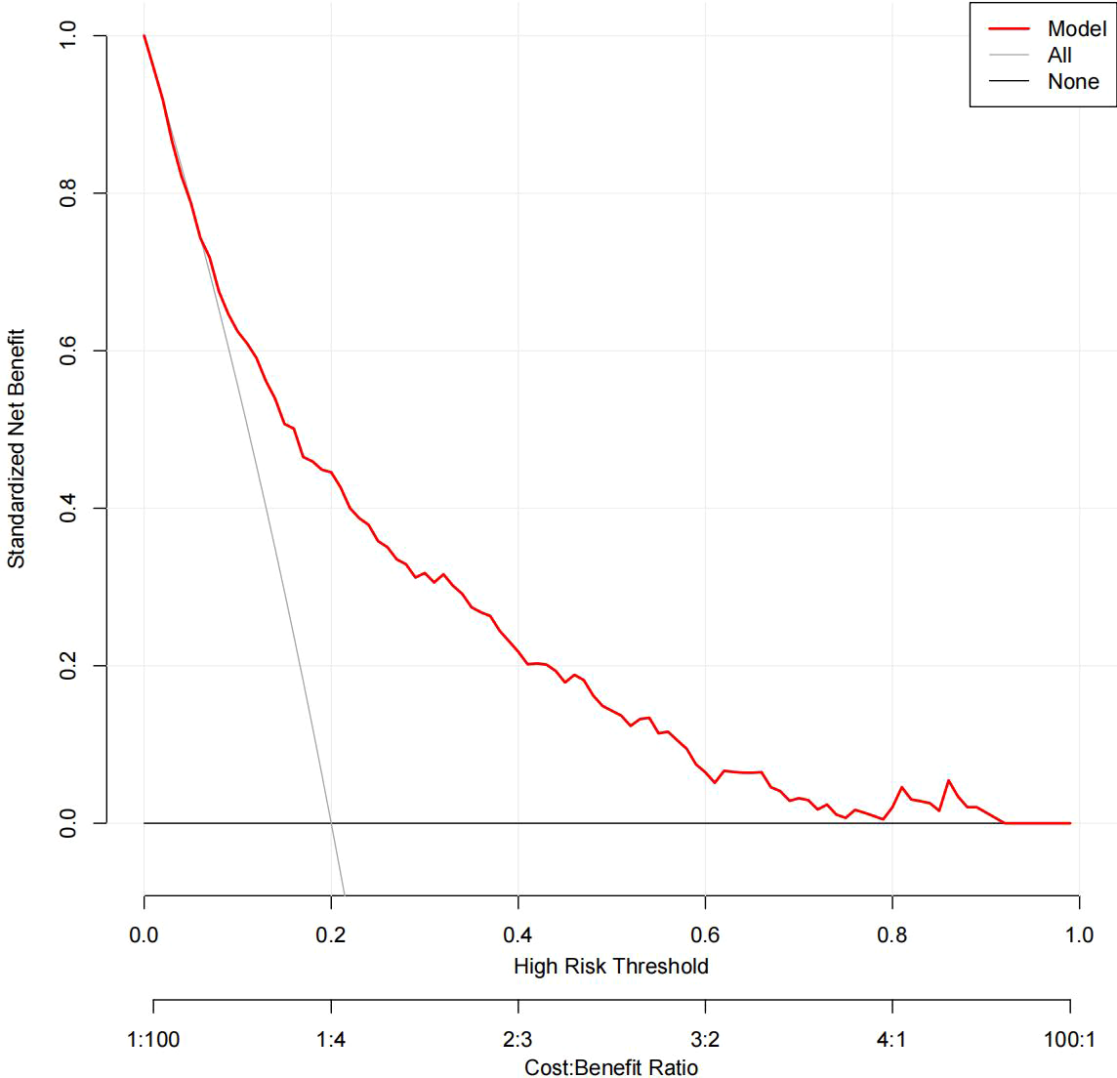
The DCA of the model using XG Boost ML. DCA, decision curve analysis; XG Boost ML, extreme gradient boosting (XG) machine learning (ML).

The DCA results for the two models are presented in **Figures 8**, **9**. Compared with treating all women and none of the women, the prediction models using LR provide a net benefit between a threshold probability of 6%–63% and 87%–90%. The DCA plot indicated good positive net benefits in the model using XG Boost ML with a threshold probability of between 5% and 92%.

## Discussion

Early screening and prediction of the likelihood of pregnant women developing GDM are imperative to the prevention and treatment of this condition (17). We compared two models and found that XG Boost ML models had better performance in terms of discrimination and achieved a larger AUC, which was as high as 0.946. Our results are concordant with a previous study showing that ML algorithms can be more accurate than traditional LR methods (18). The HL test shows that the observed probability is largely consistent with the predicted probability, which implies that both models had good calibration.

Given evidence indicates that, in the situation of no overfitting, a prediction model with a greater number of predictors has an improved prediction ability compared with a model with fewer predictors (19). Similarly, in our study, the XG Boost ML model presents 20 predictors with a higher predictive accuracy than the LR model with four predictors. Furthermore, linear models, such as LR models, highlight a clear linear contribution of each variable for GDM models, making them available for clinical implementation, whereas XG Boost ML models can weight the importance of factors and assess their complex non-linear relationships by boosting, integrating multiple factors, assess their complex non-linear relationships by boosting, and clearly demonstrate the relative contribution of each variable to GDM (18).

A recent relative study has indicated that hematologic and biochemical parameters measured during routine antenatal examination can be used in ML models to predict GDM (20). However, it has not until now been possible to weigh the relative importance of each variable. In this study we have shown that it is possible quantify the likelihood of individual independent risk factors leading to GDM. Another related study (18) developed a ML prediction model based on a large population and weighed the importance of risk factors, but there was no exploration of biomarkers in early pregnancy in this study; by contrast, this was explored in our study.

In the two models, previous GDM was the most classical predictor, and LR analysis showed that pregnant women with previous GDM are 7.8 times more likely to develop GDM (OR* *= 7.822; *p *< 0.05). Furthermore, other model studies have shown (9, 21) that previous GDM increases the risk of GDM in a current pregnancy 13.7- to 21.1-fold (*p *< 0.05). One review also found that having GDM in a previous pregnancy is the strongest risk factor for GDM, with reported recurrence rates of up to 84% (22). In addition to previous GDM, age, HbA_1c_ level, and MAP were considered independent factors for GDM in the LR analysis. Previously, age and HbA_1c_ level have been strongly associated with an elevated risk of GDM (17, 21). With increasing age, the fertility and organ function of pregnant women are reduced, and insulin sensitivity and pancreatic β-cell function are decreased, which in turn lead to insulin resistance (IR) and an increased risk of hyperglycemia. HbA_1c_ level, an identified risk factor, can diagnose the severity of GDM and reflects the average blood glucose level in the past 2 to 3 months, which is significantly related to the degree of IR (23). A previous study revealed that HbA_1c_ level is a reliable predictor of GDM(OR* *= 3.11; *p *< 0.05)and that HbA_1c_ levels are elevated in women with GDM, although still within the normal range (24), which is consistent with our results. MAP was calculated from one-third systolic blood pressure (SBP) and two-thirds diastolic blood pressure (DBP), both of which are considered to be predictors of GDM (18, 25, 26). MAP can probably predict GDM because IR is the involved in the pathogenesis of both gestational hypertension (GH) and GDM, and the level of MAP, which can reflect the severity of GH, also stimulates a certain degree of GDM (27).

Another 16 predictors, comprising pre-pregnancy BMI and 15 laboratory parameters routinely measured during antenatal assessment, were confirmed as risk factors by XG Boost ML. Pre-pregnancy BMI, despite being considered an established predictor of GDM (28), has the lowest predictive ability, probably because of the low frequency of overweight and obesity (among our sample affecting approximately 11.700% and 14.700% of women in the training and testing sets, respectively). Another explanation is that the relationship between BMI and GDM is complex, with women with GDM and a high BMI having IR and women with GDM and a low BMI having defective insulin secretion (29).

Existing studies have identified that several laboratory parameters are independent predictors of GDM, such as glycemic markers (e.g., fasting glucose and HBA_1c_ levels), alanine aminotransferase (ALT) levels, and thyroid function (levels of the thyroid hormones T_3_ and T_4_) (9, 18, 20); all of these are available clinically in the first trimester of pregnancy. The possible link between these variables and GDM could be explained by the fact that hyperglycemia can change the hemodynamics of the body, and that these variables can reflect the inflammation and immune responses that are highly associated with IR (30). Prior research has identified several blood potential biomarkers, such as platelet count, white blood cell count, and red blood cell count, which were positively correlated with the development of GDM (30). Consistent with a previous study (9), high T_3_ and low T_4_ levels were identified as being predictors of GDM in our study, strongly confirming the existence of a close relationship between thyroid function and GDM. ALT and AST (aspartate aminotransferase), as markers of hepatocellular damage, were also examined as predictors of GDM in our study. The pathogenesis of GDM is linked with IR, which may in turn be caused by mild ALT and AST elevations (15, 31). In summary, the laboratory parameters support the hypothesis that pregnancy blood routine examination is conducive to GDM screening.

## Limitations

This study has several limitations. Firstly, this study has limited sample size. Secondly, the fact is that a time external verification was used to verify the extrapolation in a single center. Lastly, there is a lack of complete data for all laboratory parameters and a comparison of multiple ML models. Variables such as clinical features and laboratory parameters are based on retrospective data from the EMRS that may have inevitable selection biases. Further multicenter prospective studies should be carried out to update and validate the models based on a large, population-based sample. Models constructed from more variables that are available from EMRS are often the most feasible option.

## Conclusion

In conclusion, a model with four predictors and using traditional LR and a model with 20 predictors and using XG Boost ML were successfully built and used to predict GDM. Compared with traditional LR, the XG Boost ML model can improve the discrimination of a prediction model for GDM and make full use of more predictors. The common laboratory parameters from pregnant women’s antenatal assessments can be used to predict the likelihood of their developing GDM.

## Data availability statement

The datasets presented in this article are not readily available because the generated datasets belong to hospital. Requests to access the datasets should be directed to XH, 731538045@qq.com.

## Ethics statement

This study was approved by the corresponding Hospital Ethics Committee (No.: NYSZYYEC20200032). The patients/participants provided their written informed consent to participate in this study. Written informed consent was obtained from the individual(s) for the publication of any potentially identifiable images or data included in this article.

## Author contributions

XH and XiaolH contributed to the conception and design of the study. XH organized the database. XH and YY performed the statistical analysis. XH wrote the first draft of the manuscript. XH, XiaolH, YY, and JW wrote sections of the manuscript. All authors contributed to the article and approved the submitted version.
